# A novel reporter of notch signalling indicates regulated and random notch activation during vertebrate neurogenesis

**DOI:** 10.1186/1741-7007-9-58

**Published:** 2011-08-31

**Authors:** Filipe Vilas-Boas, Rita Fior, Jason R Swedlow, Kate G Storey, Domingos Henrique

**Affiliations:** 1 Instituto de Medicina Molecular and Instituto de Histologia e Biologia do Desenvolvimento, Faculdade de Medicina da Universidade de Lisboa, Av Prof. Egas Moniz, 1649-028 Lisboa, Portugal; 2 Champalimaud Neuroscience Programme at Instituto Gulbenkian de Ciência, Rua da Quinta Grande, 6, 2780-156 Oeiras, Portugal; 3 Division of Cell and Developmental Biology, College of Life Sciences, University of Dundee, Dow Street, Dundee, DD1 5EH, UK

## Abstract

**Background:**

Building the complex vertebrate nervous system involves the regulated production of neurons and glia while maintaining a progenitor cell population. Neurogenesis starts asynchronously in different regions of the embryo and occurs over a long period of time, allowing progenitor cells to be exposed to multiple extrinsic signals that regulate the production of different cell types. Notch-mediated cell-cell signalling is one of the mechanisms that maintain the progenitor pool, however, little is known about how the timing of Notch activation is related to the cell cycle and the distinct modes of cell division that generate neurons. An essential tool with which to investigate the role of Notch signalling on cell by cell basis is the development a faithful reporter of Notch activity.

**Results:**

Here we present a novel reporter for Notch activity based on the promoter of the well characterised Notch target chick *Hes5-1*, coupled with multiple elements that confer instability, including a destabilized nuclear Venus fluorescent protein and the 3' untranslated region (UTR) of *Hes5-1*. We demonstrate that this reporter faithfully recapitulates the endogenous expression of *Hes5-1 *and that it robustly responds to Notch activation in the chick neural tube. Analysis of the patterns of Notch activity revealed by this reporter indicates that although Notch is most frequently activated prior to mitosis it can be activated at any time within the cell cycle. Notch active progenitors undergoing mitosis generate two daughters that both continue to experience Notch signalling. However, cells lacking Notch activity before and during mitosis generate daughters with dissimilar Notch activity profiles.

**Conclusions:**

A novel Notch reporter with multiple destabilisation elements provides a faithful read-out of endogenous Notch activity on a cell-by-cell basis, as neural progenitors progress through the cell cycle in the chick neural tube. Notch activity patterns in this cell population provide evidence for distinct Notch signalling dynamics underlying different cell division modes and for the involvement of random initiation of Notch signalling within the neuroepithelium. These findings highlight the importance of single-cell analysis in the study of the complexity of Notch activity and provide new insights into the mechanisms underlying cell fate decisions in neural progenitors.

## Background

Regulated neuron production is critical during the generation of the vertebrate nervous system. To produce the correct number and types of neurons, a population of proliferating progenitors is maintained throughout neurogenesis and several mechanisms operate to regulate their differentiation. Upon division, a neural progenitor can either give rise to two new progenitors (P-P) or two neurons (N-N), in a so-called symmetrical division, or to another progenitor and a neuron (P-N, asymmetrical division). The outcome of these divisions is controlled by both intrinsic and extrinsic cues (reviewed in [1]), and a correct balance must be achieved to allow a steady production of neurons while at the same time ensuring that enough progenitors are maintained to sustain the whole neurogenesis process. Notch signalling is one of the extrinsic mechanisms that regulate this process and it is required to maintain a population of neural progenitors throughout neurogenesis [2]. However, it remains unclear how Notch activity performs this function, i.e. whether progenitors experiencing Notch signals will adopt a P-P or P-N division mode in order to control the rate of neurogenesis.

During neurogenesis, cells committed to differentiation express Notch ligands and activate Notch signalling in neighbouring progenitors to prevent their differentiation, in a process called lateral inhibition ([3-5], reviewed in [2]). However, this inhibition is only temporary and various mechanisms collude to terminate Notch signalling in neural progenitors (reviewed in [6]); a step that might be required to allow these cells to reset their potential and enter another round of cell fate-decision. Indeed, permanent Notch activation freezes neural progenitors in an undifferentiated state, and neurogenesis only progresses when these cells are released from Notch signalling [7,8]. This implies that Notch activity in neural progenitors is highly dynamic and that cells might experience several rounds of Notch activity before becoming committed to differentiation. This view is supported by the observed fluctuations in Notch signalling, detected with a luciferase-based reporter in cultured neural progenitors [9]. However, in contrast to cells in the presomitic mesoderm (reviewed in [10-12]), neural progenitors are not synchronized, and have different levels of Notch activity that are reflected by varying levels of expression of Notch target genes [9,13,14].

The heterogeneity in the neural progenitor population, together with the dynamic nature of Notch signalling, have made it difficult to monitor Notch activity and study its relationship to the behaviour of individual cells, as they progress through the cell cycle and undergo neurogenesis. To address this, it is therefore important to develop faithful reporters for Notch signalling that can be used in time-lapse imaging; such reporters must allow detection of Notch activity from its onset and also reveal when the signalling process is switched-off to allow neurogenesis progression. To date most green fluorescent protein (GFP)-based Notch reporter systems used in the nervous system include the reporter protein and its messenger ribonucleic acid (mRNA) but do not contain protein and/or mRNA degradation signals [8,15-27]. For this reason, GFP was reported in the majority of cases to perdure in cells long after Notch signalling was extinguished. A Notch reporter with short-lived features based on a highly unstable form of luciferase has been described [28] and used to monitor Notch activity with higher accuracy in the developing nervous system [9]. However, single-cell resolution is poor with luciferase detection systems and does not allow visualization of cellular interactions or a precise correlation of Notch activity with cell cycle dynamics.

Previous Notch reporters have been generated using synthetic promoters - minimal promoters coupled to tandem repeats of CBF1/Suppressor of Hairless/Lag-1 (CSL) binding sites [8,16,23,27] - or using promoters of Notch target genes [15,17-22,24-29]. Since the synthetic CSL promoter does not reflect all Notch activity in the developing embryo [16], promoters of Notch target genes appear to be better choices. Among the known targets, *Hes1 *expression shows little overlap with Notch activity in the neural tube and its transcription in the developing neuroepithelium is unaffected by inactivation of the Notch pathway [30,31]. In contrast, *Hes5 *transcription is severely reduced in Notch mutants [30,31] and its promoter is directly regulated by Notch [32], indicating that *Hes5 *is a bona fide target of Notch activity in the developing nervous system.

In this paper, we describe the design, validation and application of a new Notch reporter system, based on the chick *Hes5 *promoter coupled to a short-lived fluorescent protein and post-transcriptional regulation signals. We show that the expression of this reporter mimics endogenous Notch activity in the neuroepithelium, providing a faithful read-out of Notch signalling. We have used this new reporter to monitor Notch activity in individual neural progenitors of the developing chick spinal cord. Our data suggest that the onset of Notch activity can be a random event and indicate a link between the neural progenitors' fate and patterns of Notch activity.

## Results

### Design of a novel reporter of Notch activity

To generate a reporter system able to monitor Notch activity during neurogenesis, a promoter that responds positively to Notch signalling in the developing nervous system must be linked to a complementary deoxyribonucleic acid (cDNA) encoding a reporter protein whose detection reflects the promoter's transcriptional activity. The chicken genome contains three *Hes5 *genes clustered in a 20 Kbp region on chromosome 21, all of which respond to Notch signalling [14]. This gene cluster is close to the *Pank4 *gene, which also flanks the single *Hes5 *gene in the mouse and human genomes [14]. In the chick, *Hes5-1 *is the gene located nearest to *Pank4*, and has the highest homology to mouse *Hes5 *(Additional file 1. Fig S1A, B), being expressed in progenitors within the ventricular zone of the neural tube [14]. Moreover, its promoter is highly similar to the human and mouse *Hes5 *promoter (53% and 58% identity, respectively, over the most proximal 400 nucleotides), containing several putative CSL-binding sites (CBS), with two highly conserved CBS in the most proximal region (Additional file 1. Fig S1C). We therefore chose a DNA fragment from the region immediately upstream of the predicted transcription initiation site of *Hes5-1 *to be used as the promoter in the Notch reporter system.

**Figure 1:**
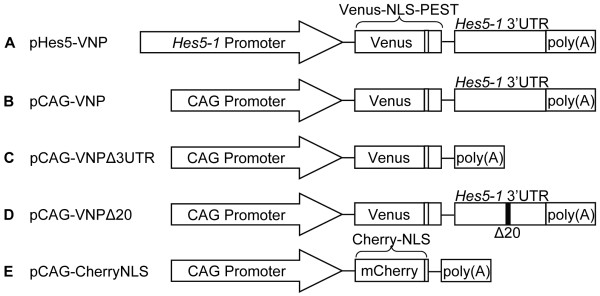
**Schematic representation of various reporter plasmid constructs generated and electroporated in the chick neural tube**. (A) pHes5-VNP, Notch reporter. (B) pCAG-VNP, reporter control. (C) pCAG-VNPΔ3UTR, reporter control without the 3'UTR of *Hes5-1*. (D) pCAG-VNPΔ20, reporter control with 20 bp missing from the 3'UTR of *Hes5-1*. (E) pCAG-CherryNLS electroporation control.

To visualize transcriptional activity mediated by the *Hes5-1 *promoter, a two kilobase pair DNA fragment upstream of the *Hes5-1 *translation start site was linked to a cDNA encoding VNP [33]. The fusion protein contains the fluorescent protein Venus - a derivative of enhanced yellow fluorescent protein (EYFP) with fast maturation and increased brightness [34] -, a nuclear localization signal (NLS) to facilitate single-cell analysis and, finally, a PEST sequence from the mouse ornithine decarboxylase protein that confers fast degradation to the fusion protein [35]. We also included the 3' untranslated region (UTR) of the *Hes5-1 *mRNA in the reporter construct, because the presence of this element further decreases the time during which the reporter is active (see below). In addition, to ensure that a poly(A) tail is present in the mRNA, we included a rabbit *β -globin *polyadenylation signal downstream of the 3'UTR. The final reporter construct (P*_Hes5-1_*-*VNP-*3'UTR*_Hes5-1_*-poly(A)) will be referred in this paper as pHes5-VNP (Figure 1A). We built also a control construct in which the *Hes5-1 *promoter is replaced by the constitutively active cytomegalovirus-actin-globin (CAG) hybrid promoter [36] - this construct (pCAG-VNP-3'UTR*_Hes5-1_*-poly(A)) will be referred as pCAG-VNP (Figure 1B).

### Expression of pHes5-VNP recapitulates the endogenous *Hes5-1 *expression

**Figure 2:**
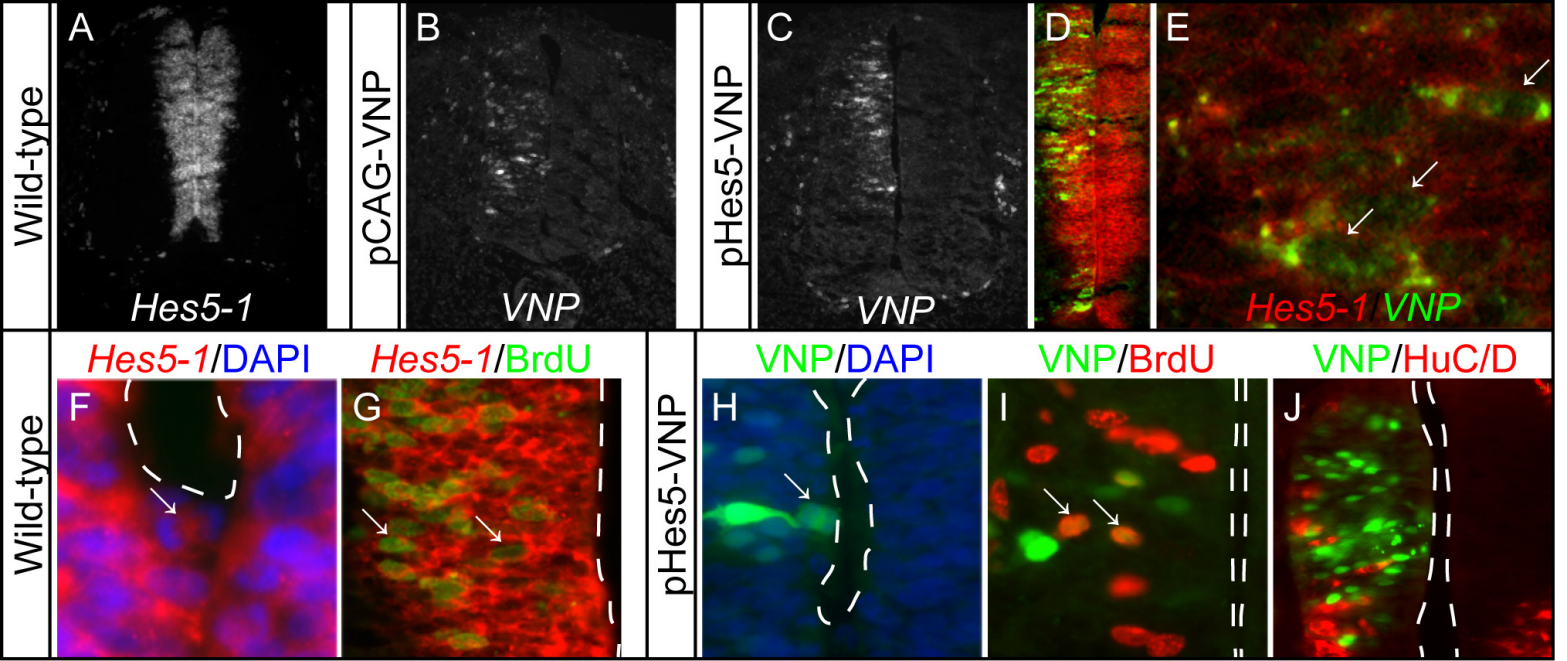
**Expression of the pHes5-VNP reporter mimics *Hes5-1 *mRNA expression**. (A-E) Following electroporation of pHes5-VNP, *VNP *mRNA can be detected only in the ventricular zone of the neural tube (C) similar to *Hes5-1 *mRNA (A) and is only detected in cells that also express *Hes5-1 *(D, E). In contrast, *VNP *mRNA expressed from pCAG-VNP is present not only in cells of the ventricular zone but also in the mantle layer (B). (F-I) VNP protein expression driven by pHes5-VNP is detected in cells that are mitotically active (H) or in S-phase, as detected by BrdU incorporation (I), similar to what is observed for *Hes5-1 *mRNA expression (F, G). Arrows in (E-I) identify cells co-labelled with the analysed markers. (J) Under conditions where low levels of reporter plasmids are electroporated (see Methods), VNP protein expressed from pHes5-VNP is restricted to neural progenitors, as virtually no co-expression with the HuC/D neuronal marker can be detected 24 h after electroporation. Dashed lines in (F-J) delineate the lumen of the neural tube.

To determine if reporter expression driven by the *Hes5-1 *promoter is able to mimic the endogenous pattern of *Hes5-1 *transcription, the pHes5-VNP and pCAG-VNP constructs were electroporated separately into the chick embryonic neural tube, and expression of *VNP *mRNA driven by each construct was compared with *Hes5-1 *mRNA expression. Embryos were harvested 48 h after electroporation and the presence of *VNP *and *Hes5-1 *mRNAs were analysed by *in situ *hybridization in sections (Figure 2A-C). These data show that *VNP *mRNA transcription driven by the *Hes5-1 *promoter is restricted to the ventricular zone and resembles the endogenous *Hes5-1 *expression pattern (Figure 2, compare A with C). In contrast, *VNP *mRNA transcription driven by a constitutive promoter (pCAG-VNP) occurs not only in the ventricular zone but also in the mantle layer (Figure 2B), showing that the specific activity of pHes5-VNP in neural progenitors is not an artefact of the electroporation procedure. To confirm further that pHes5-VNP expression occurs in cells that transcribe the *Hes5-1 *gene, double *in situ *hybridization for *VNP *and *Hes5-1 *(using a probe for the coding region and excluding the 3'UTR) was performed. Confocal analysis shows co-expression of the two mRNAs within the same cells, confirming that the reporter driven by the *Hes5-1 *promoter is active only in neural progenitors (Figure 2D, E). As observed for *Hes5-1 *mRNA (Figure 2F, G), VNP protein is expressed in proliferating cells during mitosis (Figure 2H) and during S-phase (labelled with a short 5-bromo-2'-deoxyuridine (BrdU) pulse, Figure 2I). Furthermore, at 24 h after electroporation 99% of VNP-expressing cells do not co-express the early neuronal marker HuC/D [37] (4086 VNP^+ ^cells, 143 sections, 4 embryos) (Figure 2J), indicating that VNP does not perdure in neural progenitors once these cells commit to differentiation.

Taken together, these results indicate that VNP expression driven by the *Hes5-1 *promoter faithfully recapitulates the expression of the endogenous *Hes5-1 *gene in neural progenitors and may therefore report Notch activity in this context.

### pHes5-VNP provides a read-out for Notch activity

**Figure 3:**
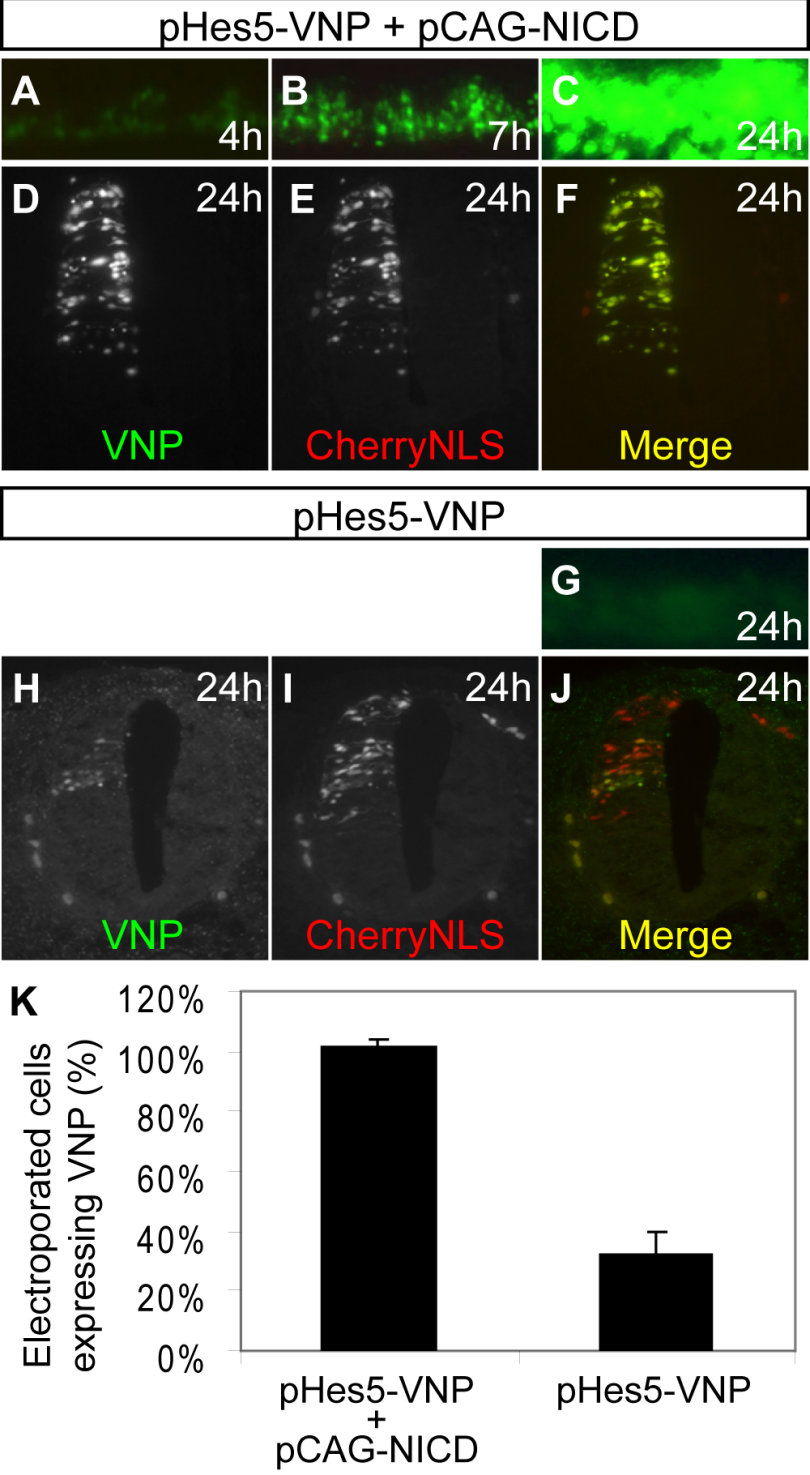
**Notch reporter's response to ectopic Notch signalling**. (A-C, G) *In vivo *time course analysis of fluorescence intensity in embryos co-electroporated with pHes5-VNP, pCAG-CherryNLS and pCAG-NICD (A-C), or pHes5-VNP and pCAG-CherryNLS (G), shows that VNP expression is higher in the presence of NICD. (D-F, H-J) Analysis of transverse sections of embryos electroporated with pHes5-VNP, pCAG-CherryNLS and pCAG-NICD (D-F), or pHes5-VNP and pCAG-CherryNLS (H-J) and harvested 24 hours post transfection, reveals that in the presence of NICD virtually all electroporated cells express VNP. (K) Chart showing the percentage of electroporated cells (CherryNLS^+^) expressing VNP under the *Hes5-1 *promoter in the absence (31.7%) or presence (~100%) of NICD.

To test the responsiveness of the pHes5-VNP reporter to Notch signalling, we monitored the appearance of VNP in conditions where the Notch pathway is ectopically activated in the chick neural tube. With this aim, a group of embryos was co-electroporated with 3 plasmids: pHes5-VNP, pCAG-NICD (a plasmid driving constitutive expression of the Notch intracellular domain) and pCAG-CherryNLS (encoding a nuclear form of a red fluorescent protein driven by the constitutive CAG promoter, allowing visualization of the nucleus of all electroporated cells, Figure 1E) (Figure 3A-F). As a control, another set of embryos was co-electroporated with pHes5-VNP and pCAG-CherryNLS, but without pCAG-NICD (Figure 3G-J).

After electroporation, the fluorescent signal in the neural tube was observed *in ovo *over time, starting at 4 hours. At this time, VNP reporter expression is detected only in embryos where NICD is ectopically expressed. VNP expression increases strongly with time and, at 24 h, embryos show very high levels of expression in the electroporated neural tube (Figure 3A-C), similar to what is observed when *Hes5-1 *mRNA expression is analysed under similar conditions [14]. In contrast, the group of embryos without ectopic Notch activation shows very weak VNP expression at 4 hours (data not shown), and the levels present at 24 h are much reduced in comparison with those elicited by NICD overexpression (Figure 3G).

**Figure 4:**
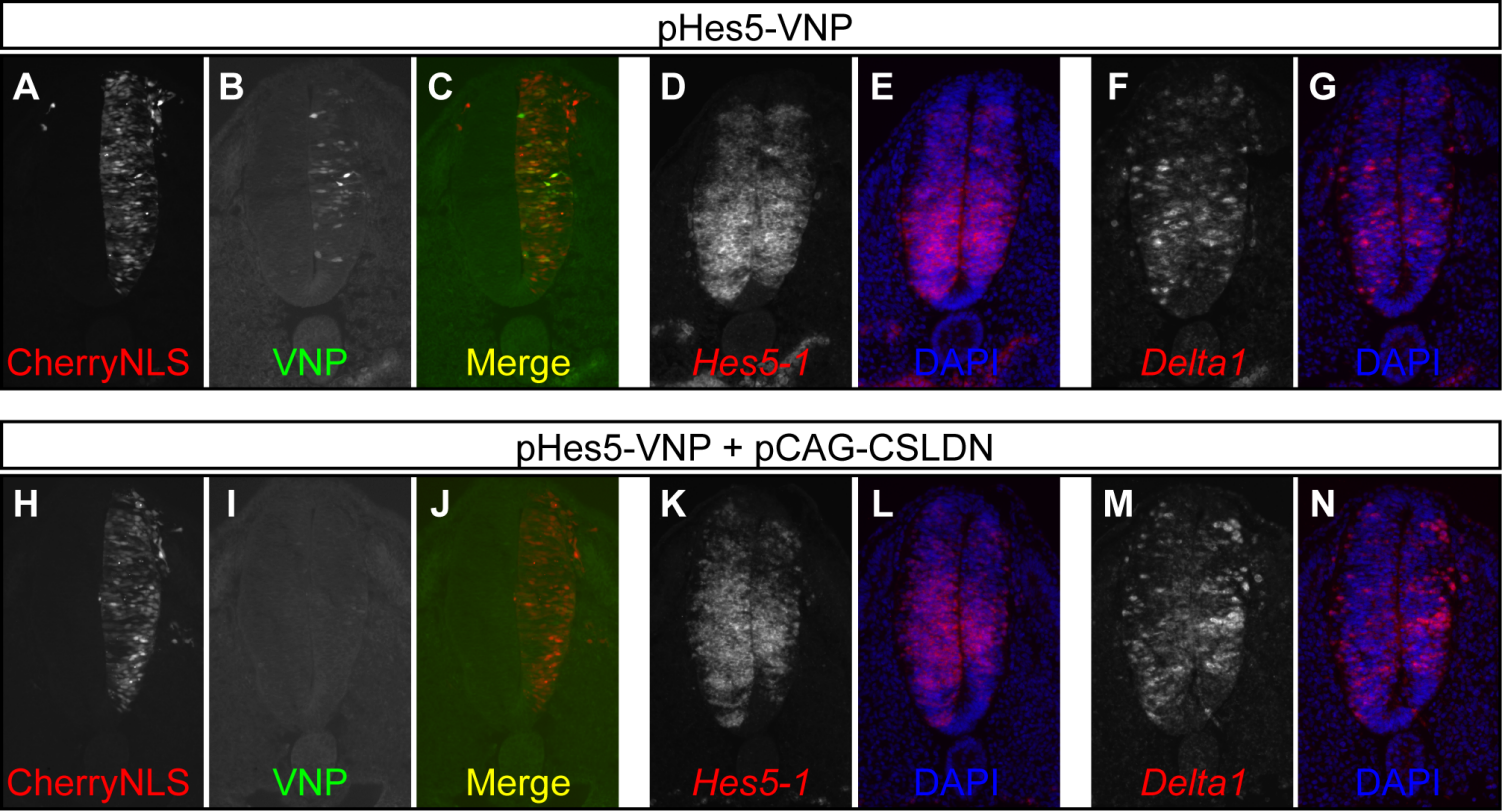
**Notch reporter's response to absence of endogenous Notch signalling**. (A-N) Analysis of transverse sections of embryos electroporated with pHes5-VNP and pCAG-CherryNLS (A-G), or pHes5-VNP, pCAG-CherryNLS and pCAG-CSLDN (H-N), and harvested 24 hours post transfection, reveals that cells where Notch signalling is abolished by misexpression of CSLDN do not express VNP. Reduction of Notch signalling in embryos electroporated with CSLDN was confirmed by observation of down-regulation of *Hes5-1 *(K-L) and up-regulation of *Delta1 *(M-N) expression. (A-C), (D-E) and (F-G) correspond to three consecutive slides of one representative embryo and (H-J), (K-L) and (M-N) correspond to three consecutive slides of another embryo. The right side of the neural tube is electroporated on all images shown.

To quantify the percentage of electroporated cells expressing the reporter in each condition, we compared the number of VNP- and CherryNLS-expressing cells in sectioned embryos, 24 h after electroporation. Without ectopic Notch activation, 31.7% ± 8.1% of the electroporated cells (CherryNLS^+^) co-express VNP (n = 806 cells, 19 sections, 3 embryos) (Figure 3H-K). This figure is similar to the number of cells that express endogenous *Hes5-1 *mRNA in the developing spinal cord of embryonic day 3 (E3) embryos (31.0% ± 6.1%; 20444 cells, 51 sections, 5 embryos), suggesting that in the absence of ectopic Notch activation, VNP expression reflects endogenous Notch activity on the *Hes5-1 *promoter present in the electroporated plasmid. By contrast, when pCAG-NICD is co-electroporated with pHes5-VNP, virtually all electroporated cells express the VNP reporter (101.40% ± 2.36%, n = 1125 cells, 17 sections, 3 embryos) (Figure 3D-F,3K). These results show that the pHes5-VNP reporter responds to ectopic Notch activity and suggests that in the absence of pCAG-NICD, reporter activity reflects endogenous Notch signalling. To confirm that reporter expression in the absence of ectopic Notch activity reflects endogenous Notch signalling, we repeated the previous experiment, but in this case quenching endogenous Notch signalling in all electroporated cells by co-electroporating pCAG-CSLDN (a plasmid driving constitutive expression of a dominant negative form of CSL). Misexpression of CSLDN has previously been shown to down-regulate endogenous *Hes5-1 *expression [14]. When Notch signalling is abolished in electroporated cells, no VNP reporter expression can be detected 24 hours post electroporation (14/14 embryos, 2 independent experiments) (Figure 4, compare H-J with A-C). In addition, pCAG-CSLDN expression results in reduced *Hes5-1 *transcription and increased *Delta1 *expression (Figure 4, compare K-N with D-G), as expected when Notch signalling is blocked in the neural tube [14]. Similar results are obtained when embryos are harvested 8 hours post electroporation, when all electroporated cells are still in the ventricular zone of the neural tube (12/12 embryos, 2 independent experiments) (data not shown). Altogether, these results confirm that the reporter system using the *Hes5-1 *promoter responds effectively and specifically to Notch signalling activity in the chick embryonic neural tube.

### The presence of the 3'UTR of *Hes5-1 *reduces expression of reporter protein

Several mechanisms may restrain the duration of Notch activity in neural progenitors, including post-transcriptional regulation of mRNA turnover by control elements in the 3'UTR, as shown for the modulation of x*Hairy2 *expression during somitogenesis in the frog [17]. To evaluate if the 3'UTR of *Hes5-1 *mRNA contributes to the post-transcriptional regulation of reporter expression, we built a derivative of the pCAG-VNP vector without the 3'UTR of *Hes5-1*, named pCAG-VNPΔ3UTR (Figure 1C). Both plasmids contain the same constitutive promoter driving *VNP *expression in all electroporated cells. We then compared the levels of VNP expression elicited by the two vectors in the chick neural tube. Each vector was co-electroporated with pCAG-CherryNLS as a control for electroporation efficiency. Embryos were collected 24 hours later and both red and yellow fluorescence intensities were analysed in whole embryos and after sectioning.

**Figure 5:**
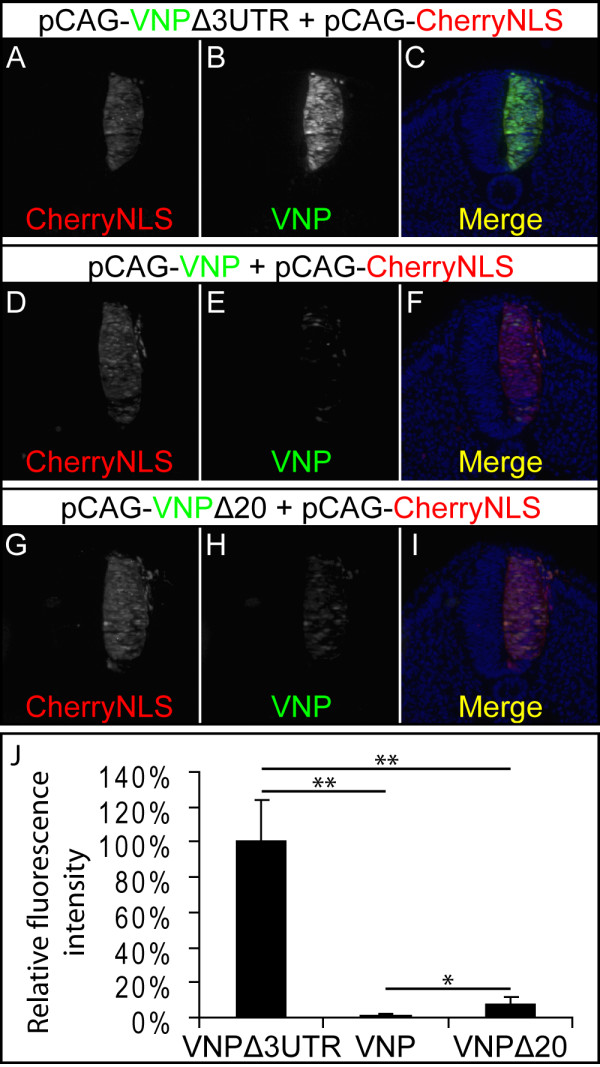
**Effect of *Hes5-1 *3'UTR on the expression of the VNP reporter protein**. (A-I) Analysis of VNP and CherryNLS expression in sections of embryos co-electroporated with either pCAG-VNPΔ3UTR and pCAG-CherryNLS (A-C), pCAG-VNP and pCAG-CherryNLS (D-F), or pCAG-VNPΔ3UTR20 and pCAG-CherryNLS (G-I), shows that the presence of the 3'UTR of *Hes5-1 *diminishes the expression of VNP and that the conserved 20 bp region in the 3'UTRs of *Hes5 *genes has a significant contribution to this effect. DAPI (blue) was used for nuclear counterstaining in C, F and I. (J) Chart showing the percentage of VNP expression from the different reporter constructs relative to that of pCAG-VNPΔ3UTR. Statistical analysis was done using t-test; *p-value < 0.002; **p-value < 0.0002.

Our results show a strong increase of VNP expression in embryos electroporated with pCAG-VNPΔ3UTR (Figure 5A-C), when compared to embryos electroporated with pCAG-VNP (Figure 5D-F). Quantification of fluorescence intensities in neural tube sections shows that electroporation with pCAG-VNPΔ3UTR results in a 99.1% increase with respect to pCAG-VNP (p-value = 0.0001, *t*-test, 32 sections, total of 4 embryos in 2 independent experiments) (Figure 5J). These results show that the presence of the *Hes5-1 *3'UTR in the pCAG-VNP vector strongly reduces the levels of reporter expression, most likely by decreasing the half-life of the VNP mRNA or by preventing translation of the reporter protein.

Sequence alignment of the 3'UTRs of mouse *Hes5*, human *Hes5 *and chick *Hes5-1 *shows only very few conserved regions, the longest spanning 20 bp (Additional file 2. Fig S2). This region contains a sequence (cTATGATa) that resembles a K-box sequence present in *Drosophila Enhancer of Split *gene transcripts (consensus sequence cTGTGATa), which has been reported to be a binding site for specific microRNAs [38,39]. To test if this conserved region is responsible for the post-transcriptional regulatory activity of the *Hes5-1 *3'UTR, we deleted it from pCAG-VNP and compared the levels of VNP expression elicited by this construct (pCAG-VNPΔ20 (Figure 1D)) to that of pCAG-VNP and pCAG-VNPΔ3UTR. The pCAG-CherryNLS vector was again used as a control for electroporation efficiency and embryos were harvested 24 h after electroporation (Figure 5G-I). Fluorescence intensities were quantified in cryostat sections. Comparison between cells electroporated with pCAG-VNPΔ20 or pCAG-VNP reveals that removal of the 20 bp sequence from the *Hes5-1 *3'UTR leads to an eight-fold increase of fluorescence levels (p-value = 0.0016, *t*-test, 32 sections, total of 4 embryos in 2 independent experiments), although still reduced in comparison to the fluorescence intensity of embryos electroporated with pCAG-VNPΔ3UTR (Figure 5J). This implies that the 20 bp conserved sequence has a significant contribution to the activity of the *Hes5-1 *3'UTR in the post-transcriptional regulation of linked mRNAs, although other regulatory elements must be present in the *Hes5-1 *3'UTR and contribute to the same end. Together, these results reveal that the *Hes5-1 *3'UTR is important to modulate the expression of the Notch reporter and its inclusion in the final construct is therefore crucial to provide a more faithful read-out of the dynamic nature of Notch signalling.

### Live-imaging of pHes5-VNP reveals distinct behaviours of Notch activity in sibling cells

The rigorous validation experiments described above establish that the *Hes5-1 *promoter provides a highly accurate read-out of Notch signalling activity, and that the fluorescent VNP protein is a suitable reporter of promoter activation. We next used this reporter system to monitor Notch activity in embryonic neural progenitors, using slices of electroporated embryos cultured in chemically defined serum-free conditions for up to 72 hours [40]. Using these conditions, neural progenitors proliferate, show clear interkinetic nuclear movement and display the ability to give rise to differentiated progeny [40]. Embryos were co-electroporated with the pHes5-VNP and pCAG-CherryNLS vectors, so that electroporated cells could be permanently traced by red fluorescence, while VNP expression reflected the dynamics of Notch signalling. In separate control experiments, another group of embryos was co-electroporated with pCAG-VNP and pCAG-CherryNLS plasmids. In this case, expression of VNP and CherryNLS is driven by the same constitutive promoter. After electroporation, slices from both groups of embryos were prepared and cultured together in the same Petri dish. Fluorescent images were taken at 7 or 10 minute intervals, using a wide-field DeltaVision imaging system as described previously [40]. Slices were incubated for up to 48 hours and imaging started at either 4 or 12 hours after electroporation. Images were processed and analysed as described in Methods. From 36 embryos electroporated with pHes5-VNP in 12 independent experiments, 704 VNP-expressing cells were analysed in 36 embryonic slices. We did prospective and retrospective studies in individual cells aiming at identifying cell divisions and thereby defining cell lineages. From the 704 VNP-expressing cells, we have defined 175 lineages, each one containing only one of the 704 VNP-expressing cells, with VNP expression occurring before and/or after mitosis. The remaining 529 VNP-expressing cells were excluded from further analyses since we could not identify the previous cell division or the next, as they became out of focus or the time-lapse imaging ended before mitosis could be observed. Thus, from this analysis, we could identify 175 lineages of VNP-expressing cells and monitor their behaviour for different periods of time.

**Figure 6:**
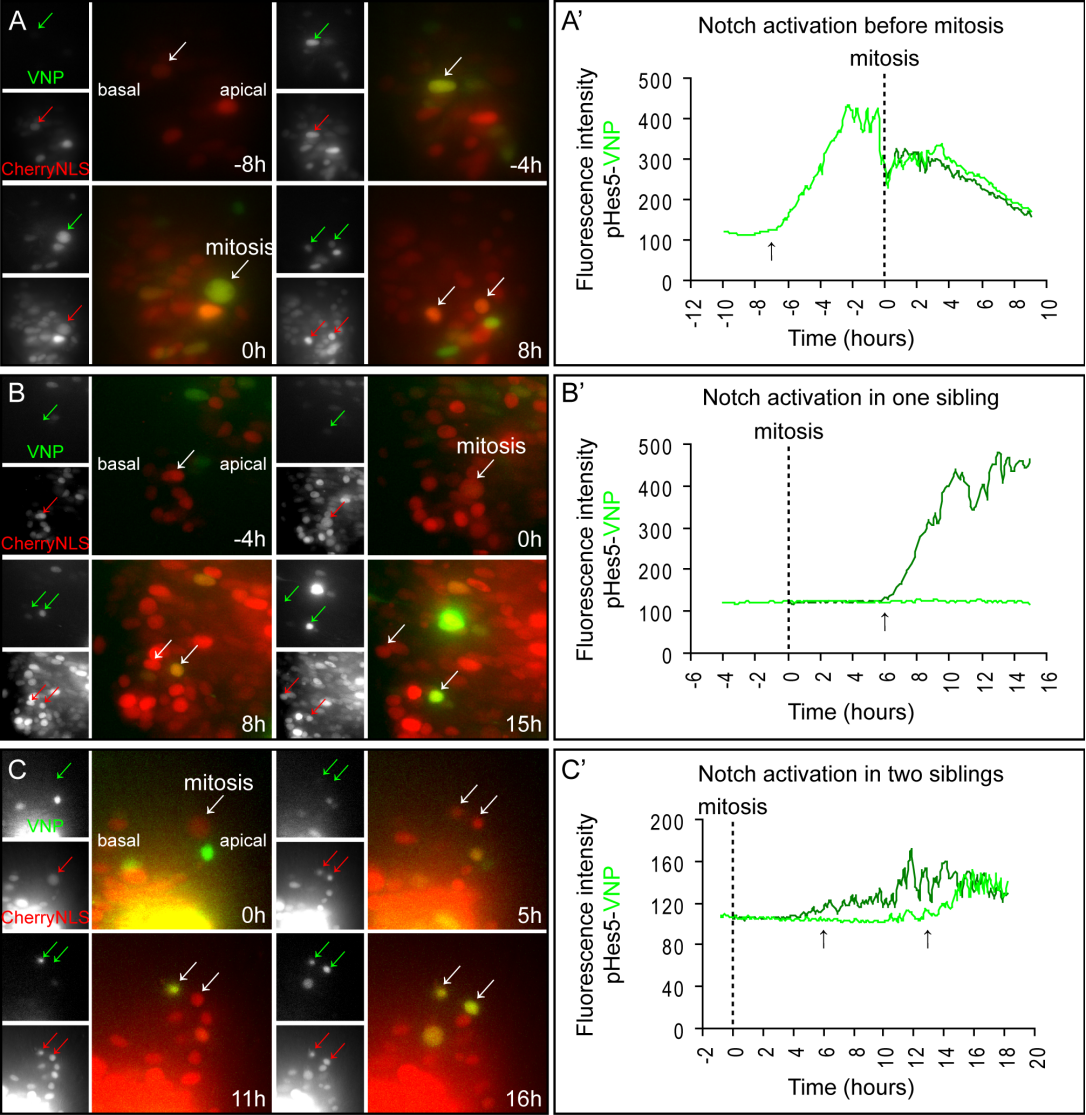
**Monitoring of Notch activity in embryos electroporated with pHes5-VNP and pCAG-CherryNLS**. (A, B, C) Neural tube slices of embryos transfected with pHes5-VNP and pCAG-CherryNLS were imaged in a wide-field microscope. The selected images in (A) show that Notch activity is detected before and during mitosis, with the two daughter cells containing similar levels of Notch activity. In (B), Notch activity is detected after mitosis in only one of the two daughter cells, while in (C) Notch becomes active after mitosis in both daughter cells, but at different times. Images presented are from maximum intensity projections. (A', B', C') Charts showing the pHes5-VNP fluorescence intensities of the (A, B, C) cell lineages along time, with mitosis as a reference point. Arrows in (A', B', C') indicate the onset of Notch activity.

**Figure 7:**
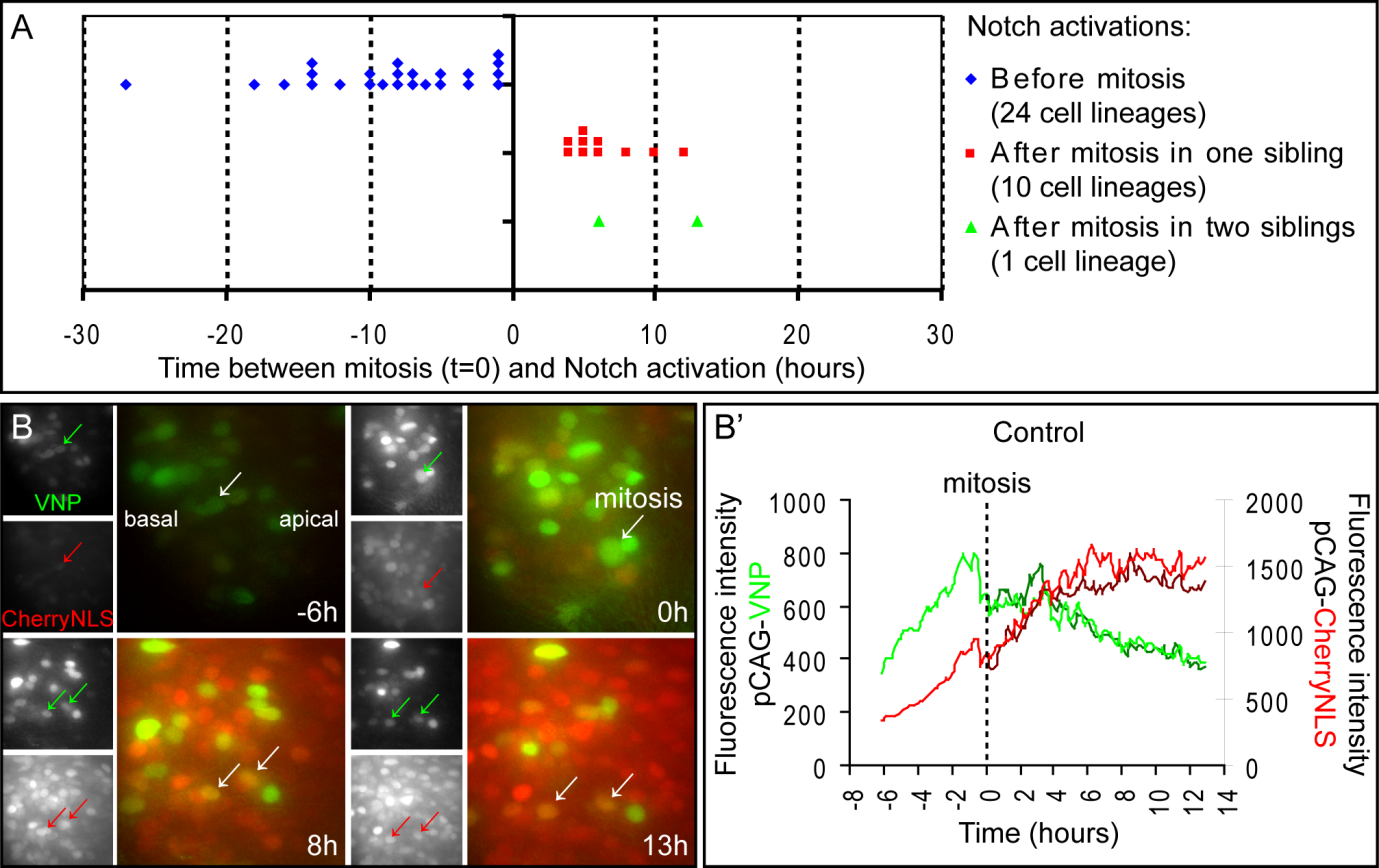
**Different onset times of Notch activity and monitoring of embryos electroporated with pCAG-VNP and pCAG-CherryNLS**. (A) Chart showing the distribution of Notch activation events in different cell lineages, indicating a wide-range of times for the onset of Notch activity during the cell cycle: 27 h to 1 h before mitosis (24 lineages), 4 h to 12 h after mitosis in only one daughter cell (10 lineages), and 6 h and 13 h after mitosis in the two daughter cells (1 lineage). Note that the sum of the number of lineages represented in the chart (35) is larger than the number of cell lineages analysed in this study (30 lineages). This is due to analysing as two separate lineages the same cellular lineage in which two mitotic events could be observed. (B) Neural tube of embryos transfected with pCAG-VNP and pCAG-CherryNLS and imaged in a wide-field microscope. An increase in VNP expression is detected soon after transfection followed by a rapid decrease of fluorescence intensity indicative of plasmid loss. In contrast, CherryNLS expression increases after transfection and persists for a longer period due to its lower instability. Images are from maximum intensity projections. (B') Chart showing the fluorescence intensities of a typical cell lineage in embryos electroporated with pCAG-VNP and pCAG-CherryNLS.

To correlate the timing of the onset of Notch activity in relation to the cell cycle, we analysed lineages where *de novo *pHes5-VNP expression could be clearly monitored (Figure 6 and 7). From the 175 VNP-expressing lineages, we could not observe *de novo *pHes5-VNP expression in 145 lineages, as these moved into the imaging planes already expressing VNP, or expressed it since the beginning of the imaging due to Notch activation soon after electroporation. We analysed instead 30 cell lineages where the timing of the onset of Notch activity could be clearly determined.

When correlated with cell cycle phase, these 30 lineages could be classified into two groups: one where Notch activation occurs before mitosis and another where activation occurs after cell division. In some cases, two mitotic events were observed within the same cellular lineage and each was analysed as a separate lineage.

In the first group, we observed that the onset of Notch activity occurs in a wide range of times before mitosis, from 1 to 27 h (24 lineages, Figure 6A, A' and Figure 7A. See also Additional file 3. Fig S3 and Additional file 4. Movie S4). In the majority of these cells (21/24), the VNP reporter is still detected in both daughter cells after cytokinesis and VNP expression is similar between siblings, suggesting that both cells experience Notch activity (Figure 6A, A'). In four of these lineages, we could observe that the two daughter cells divided again during the observation period (data not shown), revealing their progenitor character.

In the second group, containing cells in which the onset on Notch activity occurs after mitosis (11 lineages), we observed that in the majority of cases (10/11), Notch is activated in only one of the two sibling cells, an event that may occur at different times after cytokinesis, from 4 to 12 h (Figure 6B, B' and Figure 7A. See also Additional file 5. Fig S5 and Additional file 6. Movie S6). However, in one cell lineage we could detect Notch activation in the two daughter cells after mitosis, although with different onset times: one cell activating Notch 6 h after mitosis and the other 7 h later (Figure 6C, C' and Figure 7A. See also Additional file 7. Fig S7 and Additional file 8. Movie S8). These findings suggest that progenitors in which Notch is inactive during mitosis give rise to daughter cells with different potential to activate Notch signalling, or that activation of Notch signalling in this context is random (depending on their chances to contact a ligand-expressing cell). Overall, given that the average cell cycle length in chick neural progenitors is 16 hours [40], our results show that Notch signalling can be activated in different phases of the cell cycle and at a wide range of times.

We have also analysed Notch activity patterns generated by this reporter to elucidate the duration of signalling and its termination. A not infrequent event following transfection of chick embryos is plasmid loss [41,42] and this might limit the use of the reporter as an indicator of Notch activity downregulation in neural progenitors, as cessation of promoter activity cannot be easily distinguished from plasmid loss. We assessed this by following electroporated cells where CherryNLS and VNP are driven by the CAG constitutive promoter. Our results show that while the stable CherryNLS protein persists for a long time in electroporated cells, VNP levels driven by the same constitutive promoter decrease more rapidly, after the initial post-electroporation increase (Figure 7B, B'. See also Additional file 9. Fig S9 and Additional file 10. Movie S10). Such decrease in VNP expression must be due to plasmid dilution and loss from electroporated cells, followed by degradation of the remaining unstable *VN*P mRNA and VNP protein, while the more stable CherryNLS perdures. This finding therefore precludes the routine use of plasmid electroporation as an assay to monitor the termination of Notch signalling using our reporter system. However, we did observe some cells where reduction of Notch activity is followed by a new transcriptional activation of the reporter, allowing us to exclude that the previous downregulation was due to plasmid loss. These cells were included in our analysis of Notch activation events and reveal that Notch signalling dynamics can be captured using this reporter. Altogether, these results show that the electroporation-based reporter assay is suitable for the analysis of patterns of Notch activation in neural progenitors, and that the timing of onset of this activity can be correlated to the behaviour of neural progenitors after division.

## Discussion

In this study we describe the development and validation of a new Notch signalling reporter and demonstrate that this provides a faithful read-out of Notch activity onset in neural progenitors. Using this reporter we reveal dynamic alterations in Notch activity during the neural progenitor cell cycle and show that the pathway can be initiated at different phases of the cycle. Our findings indicate that when Notch activity is present at mitosis, the two daughter cells maintain similar Notch activity levels, while absence of Notch activity during mitosis correlates with divergent patterns of Notch signalling in sibling cells that may indicate the adoption of different cell fates.

### pHes5-VNP reporter is a faithful read-out of Notch activity

The Notch reporter system used in this work employs a modified form of the fluorescent protein Venus as a reporter, containing an additional nuclear localization signal to allow single-cell resolution and quantification of fluorescence intensities, and a PEST domain to confer a short half-life to the fusion protein. A similar Venus-PEST fusion protein was shown to be able to report cyclic expression of *Lfng *in the presomitic mesoderm of mouse embryos [29]. In addition, the 3'UTR of *Hes5-1 *mRNA was also included in the final transcript encoding VNP, as we showed that it contributes to temporal regulation of the reporter.

To drive expression of the reporter protein, the promoter of *Hes5-1 *was used and its behaviour in the chick embryo assessed by *in ovo *electroporation. Analysis of neuroepithelium electroporated with the pHes5-VNP plasmid revealed that *VNP *expression occurs specifically in neural progenitors at the ventricular zone. This expression is induced by ectopic Notch signalling and abolished when endogenous Notch signalling is quenched, thereby confirming that the pHes5-VNP reporter provides an accurate read-out of Notch activation. Moreover, virtually no VNP-expressing cells show co-expression of the early neuronal marker HuC/D, indicating that VNP does not perdure in neural progenitors once these cells commit to differentiation. Together, these experiments indicate that the pHes5-VNP reporter is adequate to monitor Notch signalling activity during embryonic neurogenesis.

### Post-transcriptional regulation by the 3'UTR of *Hes5-1*

We have also investigated whether the *Hes5-1 *3'UTR could affect post-transcriptional mRNA turnover, thereby contributing to regulate *Hes5-1 *expression during neurogenesis. Our results show that the presence of *Hes5-1 *3'UTR in the final mRNA encoding VNP leads to a drastic reduction in the expression levels of this protein, indicating that the 3'UTR contains signals that limit the mRNA's half-life or that restrict its translation. Furthermore, we have identified a highly conserved region present in the 3'UTRs of *Hes5 *genes that contributes to the observed reduction in VNP expression, although other regions of the 3'UTR must also be involved in this process, as the 3'UTR without this highly conserved region still leads to a reduction of VNP expression, albeit smaller. We thus propose that the 3'UTR of *Hes5-1 *is implicated in the post-transcriptional regulation of its mRNA, and this sequence was included in the pHes5-VNP Notch reporter to allow it to better reflect Notch activity. It is interesting to note that despite the inclusion of instability elements this reporter did not exhibit the very short timeframe (2-3 h) oscillations observed in mouse neural progenitors expressing a *Hes1*-driven luciferase reporter that included an ubiquitination sequence [9]. This may not be apparent in our assay, which is characterised by varied levels of plasmid transfection, or may indicate an underlying regulatory difference.

### Live-imaging of pHes5-VNP provides new insights into the mechanisms underlying cell fate decisions in neural progenitors

Previous work in different animal models has suggested regulated Notch activity during the cell cycle of neural progenitors [43-46]. In the developing zebrafish retina, live-imaging analysis of *Her4 *reporter expression has revealed a preferential activation of Notch when the nucleus of the cell is moving apically before mitosis [44]. In different neural tissues of the developing chick, including retina, spinal cord and brain, mRNAs encoding the *Notch1 *receptor and its target *Hes5-1 *were also found preferentially at the apical surface of the tissue where mitosis occurs, but not at high levels in cells undergoing S-phase [43,45]. However, the distribution of the active intracellular domain of the Notch receptor between the different cell cycle phases varies between different mouse neural tissues. In the developing brain, NICD was detected in neural progenitors in S-phase, but not in mitosis [46]. Conversely, in the developing retina, NICD was reported to be present in the apical, but not basal region [44]. In addition, in the developing mouse spinal cord, NICD was found throughout the apical-basal axis, with the exception of the ventricular surface of the neural tube [47]. These observations suggest that Notch activity might be regulated during the cell cycle of neural progenitors in a tissue- and species-specific manner. However, these studies have investigated the overall population, overlooking how Notch activity is regulated in individual cells over time.

In the present study, we have analysed how Notch activity correlates with cell cycle phases of individual cell lineages in the developing chick spinal cord. Analysis of individual cell lineages transfected with the pHes5-VNP reporter allowed us to monitor the onset of VNP reporter activity, using the moment when cells enter mitosis as a time reference. Our data reveals that Notch activity can be triggered between 1 to 27 h before cell division, or 4 to 12 h after, but not at the moment of mitosis, agreeing with previous findings showing NICD detection throughout the apical-basal axis of the developing mouse spinal cord but not adjacent to the lumen of the tube [47]. Although these observations indicate that the onset of Notch activity does not occur in cells undergoing mitosis in the developing spinal cord, we could clearly detect expression of endogenous *Hes5-1 *mRNA and pHes5-VNP reporter in neural progenitors undergoing mitosis. This suggests that *Hes5-1 *continues to be expressed after the disappearance of NICD from the nucleus of the cell. Our high resolution images have also shown expression of *Hes5-1 *mRNA in neural progenitors undergoing S-phase. Overall, our results indicate that the pathway can be activated in different phases of the cell cycle and at a wide range of times. These findings imply that Notch activity in neural progenitors is not restricted to a specific cell cycle phase and favour a model in which activation of Notch during neurogenesis is a random event, depending on chance encounters with ligand-expressing newborn neurons, while they are still in the ventricular zone of the neural tube.

Our results revealed a preferential activation of Notch in progenitors about to enter mitosis (Figure 7A), consistent with previous observations in the zebrafish retina, indicating that Notch activity seems to occur mostly when the nucleus of neural progenitors is moving apically [44]. Our results also agree with previous findings revealing heterogeneous fluctuations of Notch activity from cycle to cycle and from cell to cell [9], as this variability might reflect random events of Notch activation, but spaced in time and dependent on contact with ligand-expressing neighbouring cells.

**Figure 8:**
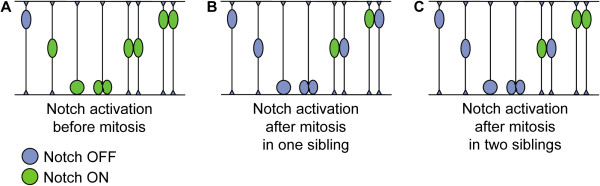
**Scheme illustrating different timings of Notch activation in cell lineages of the vertebrate neuroepithelium**. (A) When Notch is active during mitosis, the two daughter cells are born under the influence of its activity and might lead to both siblings acquiring similar fates. (B,C) When Notch becomes active after mitosis, this frequently occurs in only one of the two daughter cells (B), but it can also occur in both siblings, although at different times (C), suggesting that these cells follow distinct developmental paths. Thus, the timing of Notch activity in relation to mitosis might be a factor that distinguishes between the different fates of daughter cells.

In addition to revealing that Notch activation can occur at any time in the cell cycle, our experiments also show that when activation occurs before progenitor division, both daughter cells keep similar levels of Notch activity (Figure 8A), suggesting that they adopt the same developmental fate. We were able to confirm this in a subset of lineages in which we were able to monitor their development for a longer time. This suggestion is also supported by the higher number of progenitors in which Notch activation occurs before as opposed to after mitosis, which correlates well with the higher number of P-P compared to P-N divisions at similar stages in chick spinal cord development [40].

In contrast, when Notch activity begins after cell division, this frequently occurs in only one of the two daughter cells (Figure 8B), suggesting that these siblings now follow distinct developmental paths and might be born from a P-N division. In only one cell lineage we observed Notch activity onset occurring in two newborn siblings, although the time at which the reporter is activated differed by 7 h between the two cells (Figure 8C). Even in this case, therefore, the absence of Notch activity during cell division is correlated with different outcomes in the two sister cells.

Although we could not determine the final fate of daughter cells in all reporter-expressing lineages, our results suggest a correlation between the moment of the cell cycle when Notch is initially activated and the mode of division that follows. When a progenitor contacts a ligand-expressing neuron before mitosis, the ensuing Notch activity may restrict the neurogenic potential of the cell and lead to an obligatory P-P division. If, by chance, the progenitor does not contact a ligand-expressing cell when preparing for mitosis, the absence of Notch activity allows the progenitor to increase neurogenic competence, due to the unrestrained activity of proneural genes, and enter a neuron-producing division (P-N or N-N).

An interesting question raised by this model is how these neurogenic progenitors end up dividing symmetrically (N-N division) or asymmetrically (P-N division). The decision might be purely random, with neither of the two daughter cells receiving signals from neighbouring cells and so becoming neurons, or with one of the daughters becoming exposed, by chance, to a ligand-expressing neuron in the neighbourhood (thus acquiring a P fate), while the sister cell remains Notch-free and becomes a neuron. The two sister cells might also encounter a ligand-expressing neuron at different times, with both cells ending up activating Notch and acquiring a similar P fate. This might underlie the observed behaviour of the two sister cells that showed Notch activation with a 7-hour difference. However, this was observed only once, a finding that does not favour a simple random mechanism, as a higher number of sister cells with random times of Notch activation would be expected. An alternative mechanism to explain differential Notch activation in sister cells might involve classical Notch-mediated lateral inhibition between two daughter cells with equivalent neurogenic potential which employ competitive Notch signalling between them to acquire distinct fates (N or P), as has been described in the AC-VU decision in *Caenorhabditis elegans *[48]. Importantly, these two possibilities are not mutually exclusive. At early stages sister cells can separate quickly within the neuroepithelium and Notch activation may be random, while later on when individual neural progenitors move less in the anterior-posterior (A-P) and in the dorsal-ventral (D-V) axes interactions with sibling cells may come to predominate. The occurrence of N-N divisions, where none of the sister cells activate Notch, might arise from progenitors in which the levels of proneural gene expression are so high that this restrains both sibling cells from exchanging signals after cell division.

An additional influence that may bias Notch signalling between siblings involves the differential inheritance of apical and basal cell components determined by the cleavage plane orientation (reviewed in [1]). Progenitor divisions with cleavage planes that bisect polarized cell fate determinants might lead to symmetric N-N divisions, whereas cells with cleavage planes that bypass polarized cell fate determinants might lead to their asymmetric distribution between the two daughter cells, imposing a bias on how these will respond to Notch activating signals from neighbouring cells. In this scenario, the postulated determinants might either promote the ability of one daughter cell to activate Notch or inhibit Notch activation in the other sibling.

## Conclusions

In this study, we describe a novel Notch signalling reporter with features that allow tracking of dynamic changes in Notch activity with high resolution in single cells. Using this Notch reporter, we have monitored the timing of Notch activation throughout the cell cycle and provide new insights into the mechanisms underlying cell fate decisions in neural progenitors. This work highlights the importance of single cell analysis to study the complexity of Notch activity and neural cell fate decisions.

## Methods

### Plasmid constructs

To generate the pHes5-VNP reporter, a region of around 2Kbp upstream of the *Hes5-1 *coding region was amplified by polymerase chain reaction (PCR) from genomic DNA (Primers were 5'-CCGCTCGAGGCACACTAGGGACACTCCAGGG-3' and 5'-CATTATCCGAGAGCTGCTGTCAGC-3') and fused in frame to *Venus-NLS-PEST *(*VNP*) [33]. The 3'UTR of *Hes5-1*, amplified from an expressed sequence tag (EST) clone (GenBank Acc: BU224462, with primers 5'-CTAGTCTAGAGCCAAGAGCACGCTCACCATCAC-3' and 5'-CATTATCCGAGAGCTGCTGTCAGC-3), was then added to the P*_Hes5-1_VNP *cassette, followed by the rabbit *β-globin *polyadenylation signal to generate the final P*_Hes5-1_VNP*3'UTR*_Hes5-1_*poly(A) cassette. The Notch reporter control pCAG-VNP was generated by amplifying the *VNP*3'UTR*_Hes5-1 _*cassette from the pHes5-VNP reporter and subcloning into pCAGGS, maintaining the *Hes5-1 *Kozak sequence. pCAG-VNPΔ3UTR was obtained by subcloning *VNP *into pCAGGS. To generate pCAG-VNPΔ20, *VNP*3'UTR*_Hes5-1 _*was amplified by PCR in two fragments, which were then rejoined, leaving a 20 bp gap corresponding to the region where the putative K-box was found, and cloned into pCAGGS. pCAG-CherryNLS was cloned by annealing two complementary oligonucleotides corresponding to the Simian Virus 40 (SV40) Nuclear Localization Signal (NLS) previously used to generate VNP [33] and fusing to *mCherry *[49] in pCAGGS. A constitutively expressed active form of Notch (pCAG-NICD) was constructed by inserting a 2.6K bp DNA fragment from CNIC [50], digested with *Not*I (Fermentas, St. Leon-Rot, Germany), into pCAGGS. A constitutively expressed dominant negative form of CSL (pCAG-CSLDN) was constructed by inserting a 1.5Kbp DNA fragment from pCIG-dnSuH (kindly provided by Andy McMahon, The Biological Labs, 16 Divinity Avenue, Room 1059, Cambridge MA, 02138, USA), digested with *Xho*I/*Cla*I (Fermentas), into a modified form of pCAGGS containing the pBlueScript KS II (Stratagene, Santa Clara, CA, USA) polylinker (Bekman, E. and Henrique, D., unpublished). Detailed cloning procedures are available upon request.

### Embryo electroporation

For all analyses, with the exception of live-imaging experiments and Figure 2J, super-coiled plasmids encoding VNP were injected into the neural tube of chicken embryos staged HH11-HH13 (Hamburger and Hamilton stages [51]) (fertilized eggs provided by Sociedade Agrícola Quinta da Freiria, S.A., Roliça, Portugal and Winter Farm, Hertfordshire, UK) at a concentration of 1 μg/μl in phosphate buffered saline (PBS, Sigma, Steinheim, Germany). In some cases, pCAG-CherryNLS DNA was co-injected at a concentration of 0.2 or 1 μg/μl and pCAG-NICD or pCAG-CSLDN were injected at concentrations of 1 to 2 μg/μl. Platinum electrodes (CUY613P5, NEPA GENE, Ichikawa, Japan), distanced 4 mm apart, were placed parallel to the neural tube under the vitelline membrane. Using an Electro Square Porator™ ECM830 (BTX, Holliston, MA, USA), 4 pulses of 30V were applied for 50 ms. Embryos were incubated for 8 h, 24 h or 48 h and then harvested and fixed overnight in 4% paraformaldehyde (Sigma) in PBS at 4°C.

For live-imaging experiments and in the experiments shown in Figure 2J, super-coiled DNA encoding VNP was injected into the neural tube of chicken embryos staged HH8-HH11 at a concentration of 0.6 μg/μl in PBS. pCAG-CherryNLS was co-injected at a concentration of 0.06 μg/μl. Gold plated electrodes (Genetrode 512, BTX), distanced 4 mm apart, were placed parallel to the neural tube above the vitelline membrane and few drops of PBS were added. Electroporation was done with three pulses of 10V for 50 ms. For sectioning of fixed tissue shown in Figure 2J, embryos were incubated at 38°C for 24 h and then harvested and processed as described above. For live-imaging experiments, embryos were incubated at 38°C for 4 h or 12 h and then harvested and processed as described below.

### Embryo slice preparation, culture and imaging

After harvesting, one embryonic slice from each electroporated embryo was prepared, cultured for live-imaging and imaged as described in [40], with modifications. Embryo slices were cultured in serum-free medium with chemically defined supplements: Neurobasal medium without phenol red (Gibco, Paisley, UK), supplemented with B-27 (Gibco) to a final 1X concentration with L-glutamine (Gibco) and gentamycin (Invitrogen, Paisley, UK). Slices were imaged on a wide-field DeltaVision Spectris microscope workstation (Applied Precision, Issaquah, WA, USA) in a humidified chamber kept at 37°C. Images were captured using a 40x oil objective lens (1.35NA, Olympus, Hamburg, Germany). Thirty optical sections (50-100 ms exposure time, 512x512 pixels, binning = 2), spaced by 1.5 μm, were imaged at 7 or 10min intervals for up to 48 h. The point-visiting function in the SoftWorX software (Applied Precision) allowed up to nine slices to be imaged during each experiment. Slices electroporated with the Notch reporter pHes5-VNP were imaged simultaneously with slices electroporated with the pCAG-VNP control. After image acquisition the data was deconvolved as described [40].

### Measurement of fluorescence intensities from live-imaging experiments

Analysis of deconvolved data acquired from live-imaging experiments using the DeltaVision Spectris workstation was performed using SoftWorX, Omero (http://www.openmicroscopy.org/site/products/omero), ImageJ (http://rsbweb.nih.gov/ij), and Excel (Microsoft, Redmond, WA, USA). Individual cells were manually identified in Omero in maximum intensity projections generated in SoftWorX. Cells that expressed VNP and divided during the imaging period were selected for further analysis in non-projected deconvolved data. These cells were manually tracked in Omero by identifying the Z plane presenting the highest fluorescence intensity at each time point. A circle of defined size was then delineated in each nucleus in the chosen Z plane, and the levels of red and yellow fluorescence were quantified in Omero by measuring the mean fluorescent intensities in the outlined nucleus area of the selected frames. This process was repeated for each time frame and the levels of CherryNLS and VNP expression were plotted against time in Microsoft Excel, covering at least one mitotic event, thereby allowing the definition of cell lineages with different VNP expression patterns. Still images and movies presented correspond to maximum intensity projections generated in SoftWorX and converted to TIFF and AVI files in ImageJ.

### Measurement of fluorescence intensities from the studies of the *Hes5-1 *3'UTR

Images of embryonic sections were used to quantify VNP expression in embryos electroporated with pCAG-VNP, pCAG-VNPΔ3UTR and pCAG-VNPΔ20. Areas where the neural tube showed extensive and homogeneous signal for CherryNLS were selected. Mean fluorescence intensities of both red (CherryNLS) and yellow (VNP) channels were quantified in ImageJ and the background signal was subtracted. To normalize VNP expression to the electroporation efficiency (measured by CherryNLS expression), the ratio of yellow/red fluorescence intensities was obtained. Relative fluorescence intensities of embryos electroporated with pCAG-VNPΔ3UTR, pCAG-VNP or pCAG-VNPΔ20 were compared to the intensity of embryos electroporated with pCAG-VNPΔ3UTR.

### *In situ *hybridization and immunohistochemistry

For hybridization on cryostat sections, fixed embryos were cryoprotected in 30% sucrose (Sigma) in PBS, embedded in a solution containing 7.5% gelatine (Sigma) and 15% sucrose in PBS, frozen and cryosectioned (12 μm). Hybridization on cryostat sections was done as previously described [52], with modifications. Double *in situ *hybridization on cryostat sections was done with Digoxigenin (Dig)- and Fluorescein (Fluo)-labeled RNA probes. The Dig-labeled probe was first detected with Alkaline Phosphatase (AP)-conjugated anti-Dig antibody (1:2000, Roche, Mannheim, Germany) and developed with Fast Red substrate (Roche). After washing in PBS, sections were blocked and incubated with Horseradish Peroxidase (HRP)-conjugated anti-Fluo antibody (1:1000, Roche), followed by fluorescein isothiocyanate (FITC)-Tyramide amplification, as recommended by the manufacturer (TSA™-Plus Fluorescein System Kit, PerkinElmer, Waltham, MA, USA). When *in situ *hybridizations were performed to detect *VNP *mRNA, the electroporated plasmids were removed with DNAseI (Roche) to prevent recognition of DNA by the RNA probe [53]. To generate *VNP *or *Hes5-1 *coding region probes, *Venus-NLS-PEST *[33] or *Hes5-1 *coding region [14] were subcloned in pBlueScript KS II and used to transcribe the RNA probes. Additionally, *Hes5-1 *and *Delta1 *[4] probes were also transcribed from templates generated by PCR and containing the respective coding regions. For BrdU treatment, 100 μL (12.5 mg/mL, Sigma) were applied onto embryonic day 4 (E4) chick embryos, which were harvested and fixed 30 minutes later. Antigen retrieval for BrdU treated embryos was done using HCl 2N (Merck, Darmstadt, Germany) for 30 minutes at 37°C. Primary antibodies used were mouse anti-BrdU (1:1000, Sigma) and mouse anti-HuC/D (1:500, Molecular Probes, Invitrogen). Detailed protocols are available upon request. Images were taken with a DC350F camera (Leica, Wetzlar, Germany) on the fluorescent microscope DM5000B (Leica), with the DeltaVision Spectris workstation, or with the Carl Zeiss 510 confocal microscope (Zeiss, Welwyn Garden City, UK).

## Authors' contributions

FVB and RF participated in the design of the study, generation of plasmid constructs, chick embryo electroporations, *in situ *hybridizations, immunohistochemistry, sequence alignments and analysis, and drafted the manuscript. FVB also performed the live-imaging experiments, measurement of fluorescence intensities and data analysis. JRS provided technical expertise in live-imaging experiments and data analysis. KGS and DH conceived the study, participated in its design and coordination, and wrote the manuscript. All authors read and approved the final manuscript.

## Supplementary Material

Additional file 1 **Comparison of HES5 protein and promoter sequences**. (A, B) Analysis of sequence similarity between the different HES5 proteins (A), and the corresponding phylogenetic tree (B), shows that among all three chick HES5 proteins, HES5-1 has the highest degree of homology with mammalian HES5. (C) Sequence alignment of the proximal 400 bp promoter regions of several *Hes5*-like genes. Shaded areas represent regions of homology and highlight several conserved regions, such as TATA box, CCAAT box and CSL binding sites. The two identified high-affinity CSL-binding sites have been previously reported to be essential for Notch-mediated promoter activity [15,22,26,54]. The *Hes5-1 *promoter has the highest homology to mammalian *Hes5 *promoters and was included in the pHes5-VNP reporter. Click here for file

Additional file 2 **Comparison of *Hes5 *3'UTR sequences**. (A) Comparison of 3'UTR sequences from c*Hes5-1*, m*Hes5 *and h*Hes5*. Shaded areas represent regions of homology. The 20 bp region deleted to generate pCAG-VNPΔ3UTR20 is identified. Click here for file

Additional file 3 **Behavior of cells with Notch reporter activity prior to mitosis**. Selected set of images taken at 1 hour intervals from neural tube slices of embryos electroporated with pHes5-VNP and pCAG-CherryNLS and imaged in a wide-field microscope. This selected set of images refers to the cell described in Figure 6A showing Notch activity starting before mitosis, with the two daughter cells being born in the presence of similar levels of Notch activity. Images presented are from maximum intensity projections. Click here for file

Additional file 4 **Behavior of cells with Notch reporter activity prior to mitosis**. This movie refers to cell depicted in Figure 6A and Additional file 3. Blue dots identify the analysed cell lineage. The basal side of the neural tube is left, apical is right. Click here for file

Additional file 5 **Notch activation after mitosis in only one daughter cell**. Selected set of images taken at 1 hour intervals from neural tube slices of embryos electroporated with pHes5-VNP and pCAG-CherryNLS and imaged in a wide-field microscope. This selected set of images refers to the cell described in Figure 6B showing Notch activity starting after mitosis in only one of the two daughter cells. Images presented are from maximum intensity projections. Click here for file

Additional file 6 **Notch activation after mitosis in only one daughter cell**. This movie refers to cell depicted in Figure 6B and Additional file 5. Blue dots identify the analysed cell lineage. The basal side of the neural tube is left, apical is right. Click here for file

Additional file 7 **Notch activation after mitosis at different times in daughter cells**. Selected set of images taken at 1 hour intervals from neural tube slices of embryos electroporated with pHes5-VNP and pCAG-CherryNLS and imaged in a wide-field microscope. This selected set of images refers to the cell described in Figure 6C showing Notch activity starting after mitosis in both daughter cells, but at different times. Images presented are from maximum intensity projections. Click here for file

Additional file 8 **Notch activation after mitosis at different times in daughter cells**. This movie refers to cell depicted in Figure 6C and Additional file 7. Blue dots identify the analysed cell lineage. The basal side of the neural tube is left, apical is right. Click here for file

Additional file 9 **Differential protein stability is evident on comparison of VNP and CherryNLS expression**. Selected set of images taken at 1 hour intervals from neural tube slices of embryos electroporated with pCAG-VNP and pCAG-CherryNLS and imaged in a wide-field microscope. This selected set of images refers to the cell described in Figure 7B. Images presented are from maximum intensity projections. Click here for file

Additional file 10 **Differential protein stability is evident on comparison of VNP and CherryNLS expression**. This movie refers to cell depicted in Figure 7B and Additional file 9. Blue dots identify the analysed cell lineage. The basal side of the neural tube is left, apical is right. Click here for file
