# Supplementary figures and images for: A novel reporter of notch signalling indicates regulated and random notch activation during vertebrate neurogenesis
Source: BMC Biol. 2011 Aug 31;9:58. doi: 10.1186/1741-7007-9-58 (PMC3201213; doi:10.1186/1741-7007-9-58)

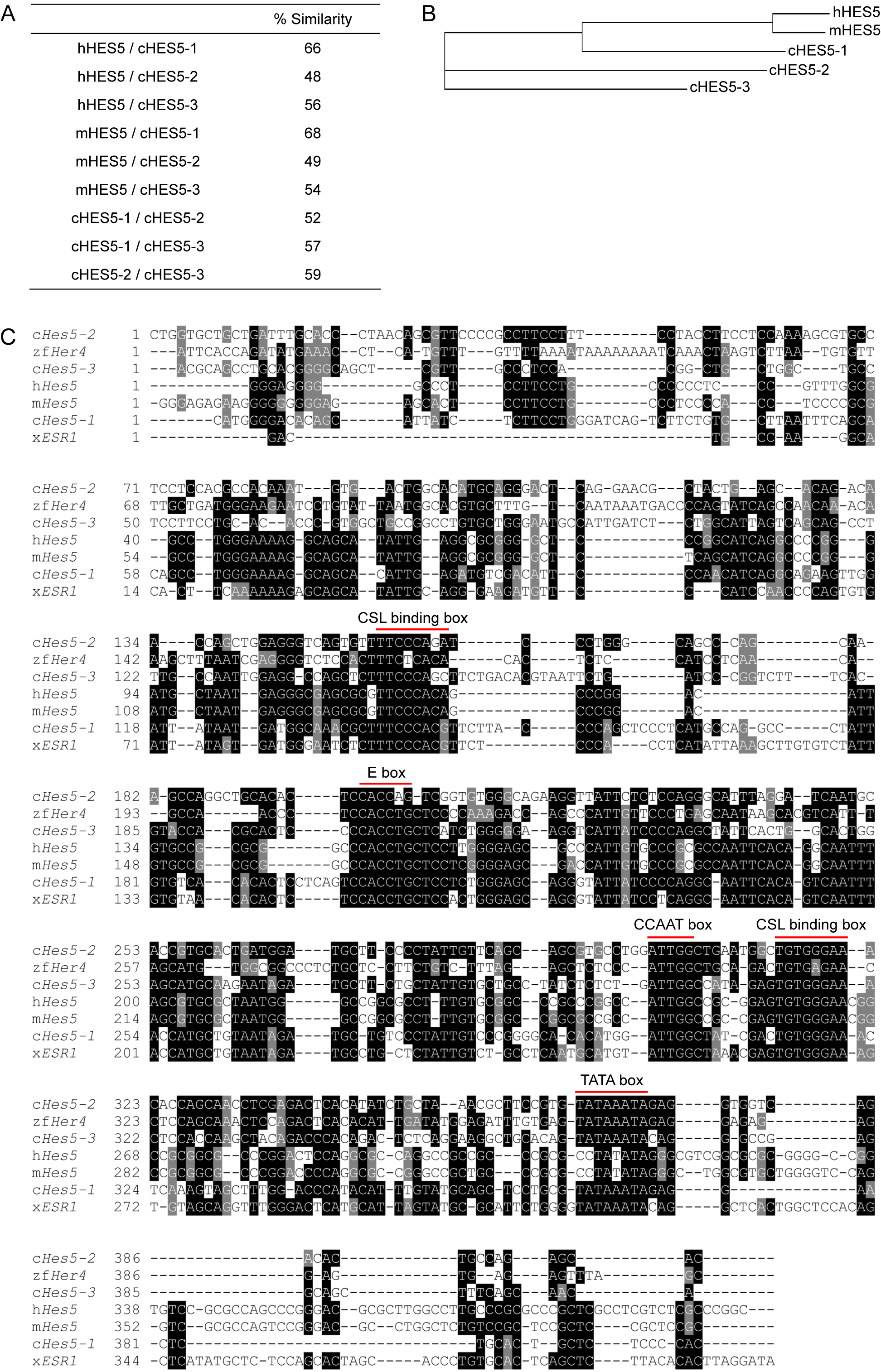

Supplement: Additional file 1 — Comparison of HES5 protein and promoter sequences. (A, B) Analysis of sequence similarity between the different HES5 proteins (A), and the corresponding phylogenetic tree (B), shows that among all three chick HES5 proteins, HES5-1 has the highest degree of homology with mammalian HES5. (C) Sequence alignment of the proximal 400 bp promoter regions of several Hes5-like genes. Shaded areas represent regions of homology and highlight several conserved regions, such as TATA box, CCAAT box and CSL binding sites. The two identified high-affinity CSL-binding sites have been previously reported to be essential for Notch-mediated promoter activity [15,22,26,54]. The Hes5-1 promoter has the highest homology to mammalian Hes5 promoters and was included in the pHes5-VNP reporter. [file 1741-7007-9-58-S1.TIFF]

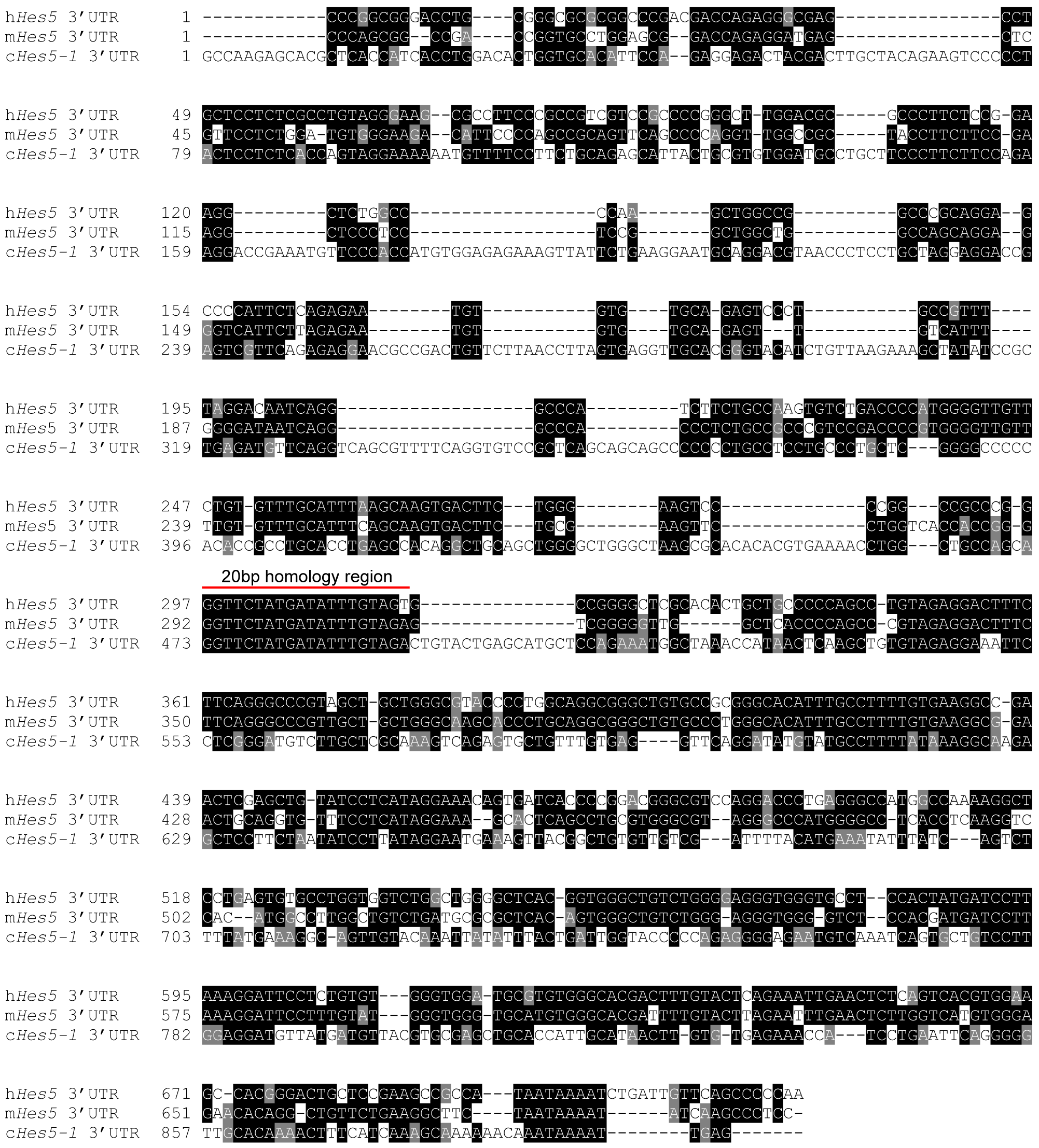

Supplement: Additional file 2 — Comparison of Hes5 3'UTR sequences. (A) Comparison of 3'UTR sequences from cHes5-1, mHes5 and hHes5. Shaded areas represent regions of homology. The 20 bp region deleted to generate pCAG-VNPΔ3UTR20 is identified. [file 1741-7007-9-58-S2.TIFF]

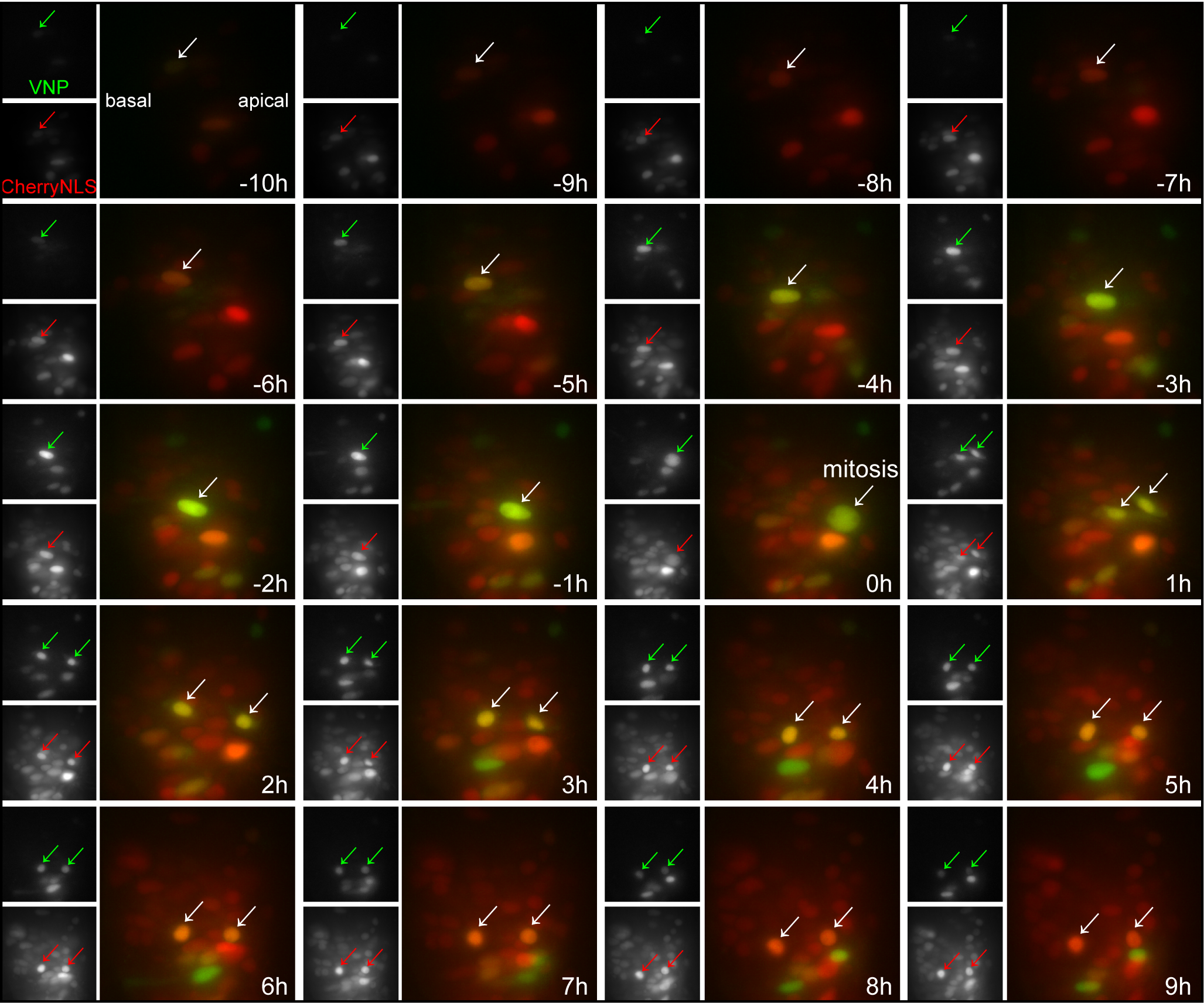

Supplement: Additional file 3 — Behavior of cells with Notch reporter activity prior to mitosis. Selected set of images taken at 1 hour intervals from neural tube slices of embryos electroporated with pHes5-VNP and pCAG-CherryNLS and imaged in a wide-field microscope. This selected set of images refers to the cell described in Figure 6A showing Notch activity starting before mitosis, with the two daughter cells being born in the presence of similar levels of Notch activity. Images presented are from maximum intensity projections. [file 1741-7007-9-58-S3.TIFF]

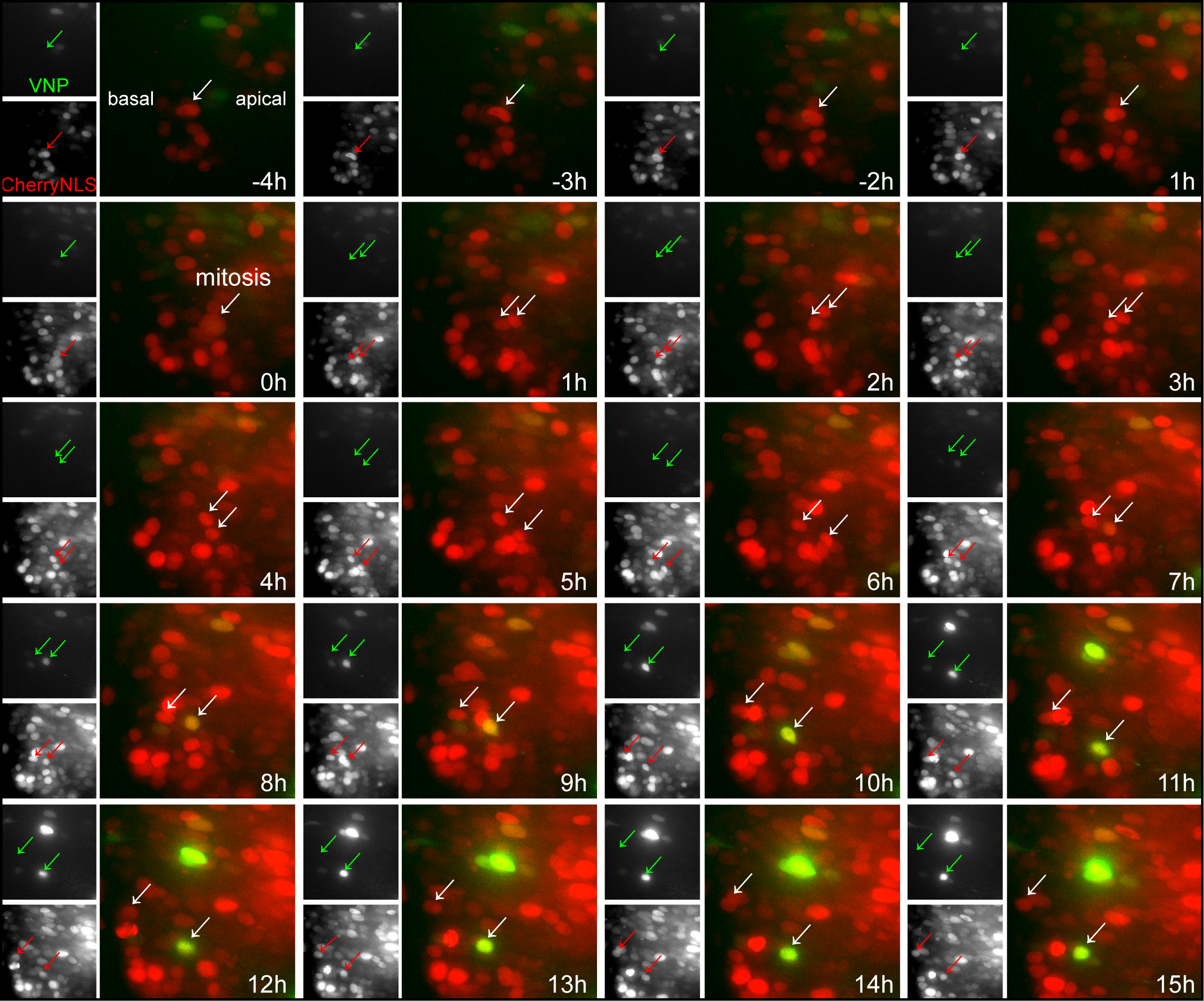

Supplement: Additional file 5 — Notch activation after mitosis in only one daughter cell. Selected set of images taken at 1 hour intervals from neural tube slices of embryos electroporated with pHes5-VNP and pCAG-CherryNLS and imaged in a wide-field microscope. This selected set of images refers to the cell described in Figure 6B showing Notch activity starting after mitosis in only one of the two daughter cells. Images presented are from maximum intensity projections. [file 1741-7007-9-58-S5.TIFF]

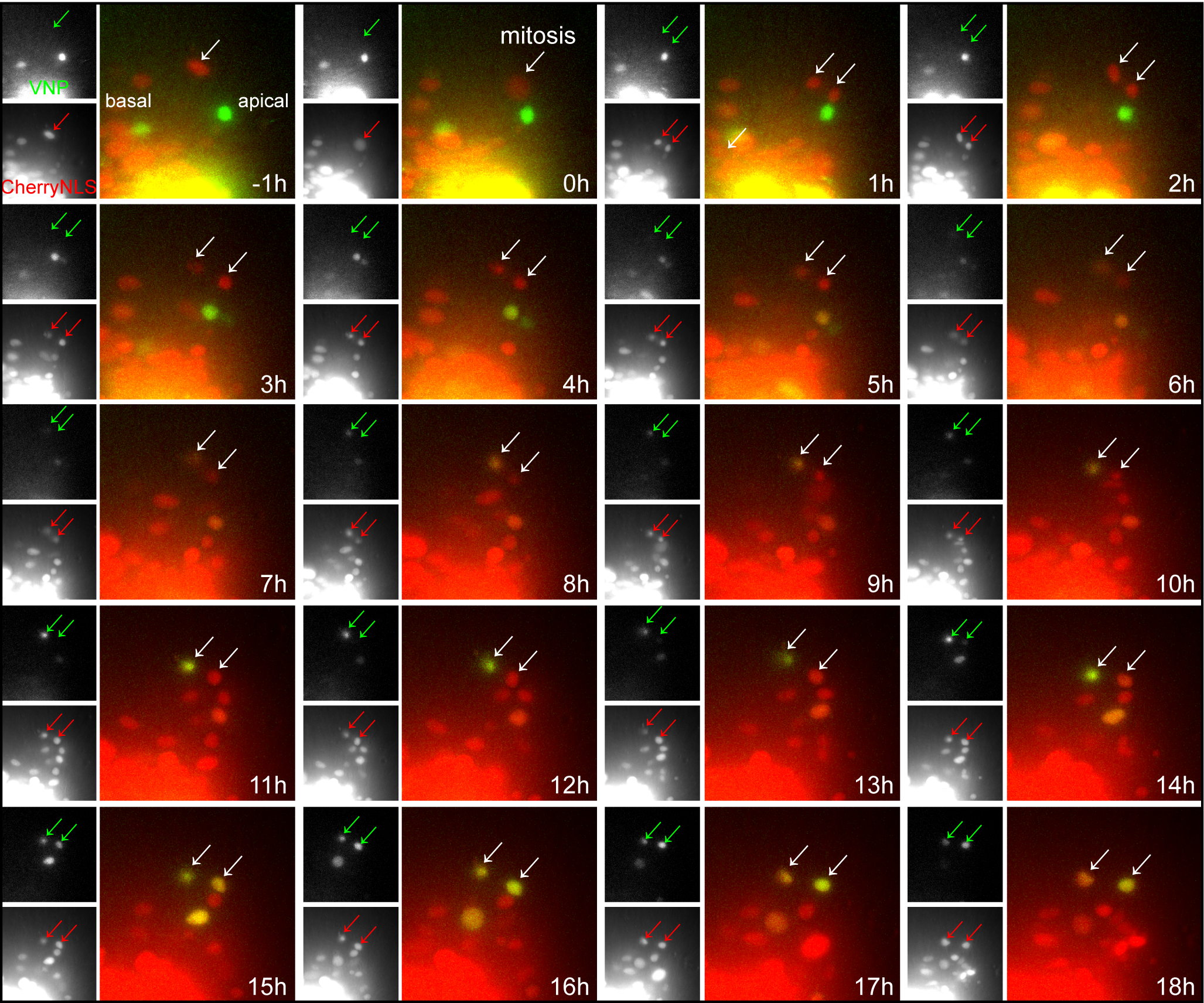

Supplement: Additional file 7 — Notch activation after mitosis at different times in daughter cells. Selected set of images taken at 1 hour intervals from neural tube slices of embryos electroporated with pHes5-VNP and pCAG-CherryNLS and imaged in a wide-field microscope. This selected set of images refers to the cell described in Figure 6C showing Notch activity starting after mitosis in both daughter cells, but at different times. Images presented are from maximum intensity projections. [file 1741-7007-9-58-S7.TIFF]

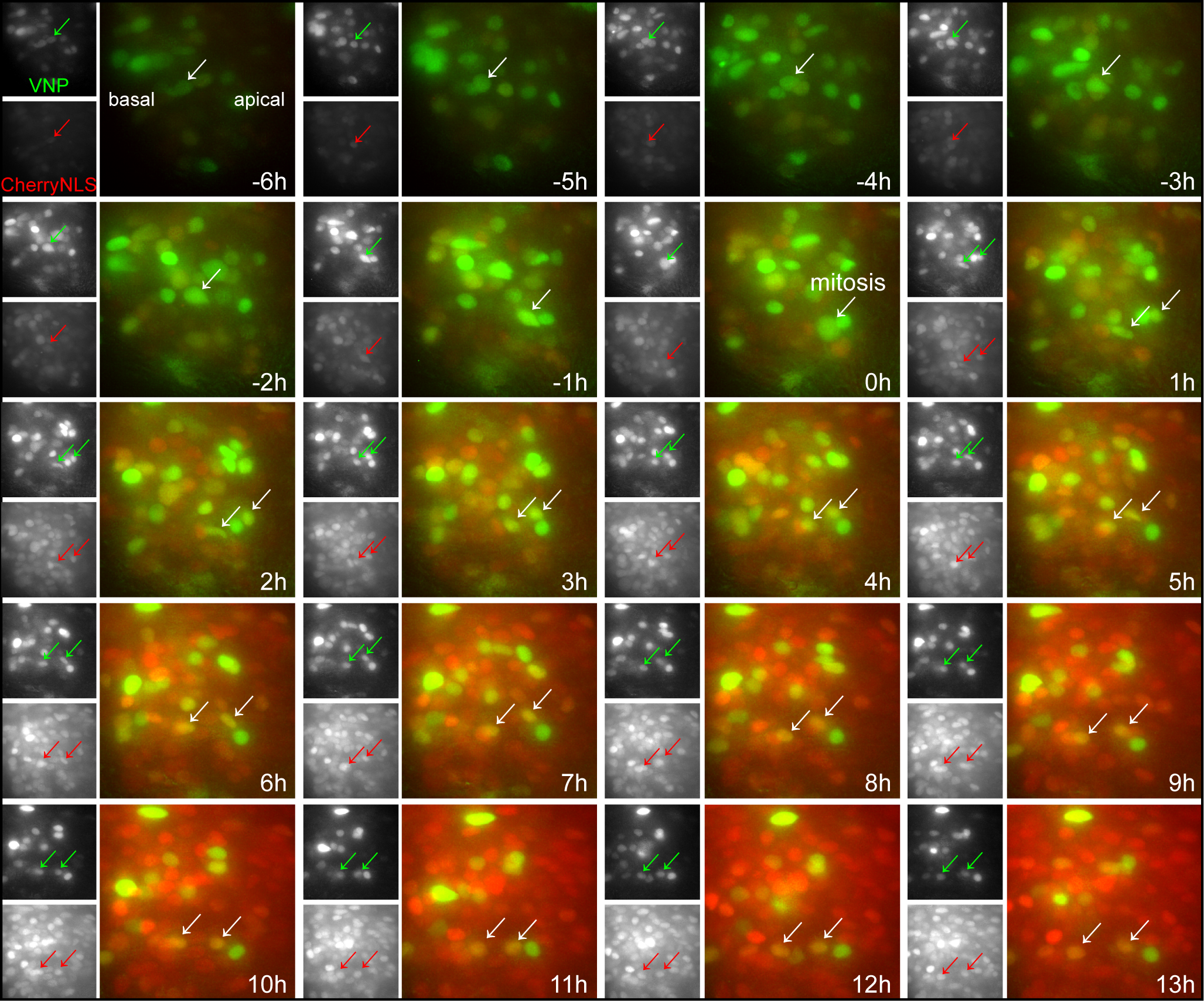

Supplement: Additional file 9 — Differential protein stability is evident on comparison of VNP and CherryNLS expression. Selected set of images taken at 1 hour intervals from neural tube slices of embryos electroporated with pCAG-VNP and pCAG-CherryNLS and imaged in a wide-field microscope. This selected set of images refers to the cell described in Figure 7B. Images presented are from maximum intensity projections. [file 1741-7007-9-58-S9.TIFF]
